# Mutations in *RHOT1* Disrupt Endoplasmic Reticulum–Mitochondria Contact Sites Interfering with Calcium Homeostasis and Mitochondrial Dynamics in Parkinson's Disease

**DOI:** 10.1089/ars.2018.7718

**Published:** 2019-10-17

**Authors:** Dajana Grossmann, Clara Berenguer-Escuder, Marie Estelle Bellet, David Scheibner, Jill Bohler, Francois Massart, Doron Rapaport, Alexander Skupin, Aymeric Fouquier d'Hérouël, Manu Sharma, Jenny Ghelfi, Aleksandar Raković, Peter Lichtner, Paul Antony, Enrico Glaab, Patrick May, Kai Stefan Dimmer, Julia Catherine Fitzgerald, Anne Grünewald, Rejko Krüger

**Affiliations:** ^1^ Luxembourg Centre for Systems Biomedicine (LCSB), University of Luxembourg, Esch-sur-Alzette, Luxembourg.; ^2^ Department of Neurodegenerative Diseases, Center of Neurology and Hertie-Institute for Clinical Brain Research, University of Tübingen, Tübingen, Germany.; ^3^ Interfaculty Institute of Biochemistry (IFIB), University of Tübingen, Tübingen, Germany.; ^4^ National Biomedical Computation Resource, University of California San Diego, La Jolla, California.; ^5^ Centre for Genetic Epidemiology, Institute for Clinical Epidemiology and Applied Biometry, University of Tübingen, Tübingen, Germany.; ^6^ Institute of Neurogenetics, University of Lübeck, Lübeck, Germany.; ^7^ Institute of Human Genetics, Helmholtz Zentrum München GmbH, Neuherberg, Germany.; ^8^ Parkinson Research Clinic, Centre Hospitalier de Luxembourg (CHL), Luxembourg, Luxembourg.

**Keywords:** Miro1, mitochondria, calcium, ER–mitochondria contact site, Parkinson's disease, patient fibroblasts

## Abstract

***Aims:*** The outer mitochondrial membrane protein Miro1 is a crucial player in mitochondrial dynamics and calcium homeostasis. Recent evidence indicated that Miro1 mediates calcium-induced mitochondrial shape transition, which is a prerequisite for the initiation of mitophagy. Moreover, altered Miro1 protein levels have emerged as a shared feature of monogenic and sporadic Parkinson's disease (PD), but, so far, no disease-associated variants in *RHOT1* have been identified. Here, we aim to explore the genetic and functional contribution of *RHOT1* mutations to PD in patient-derived cellular models.

***Results:*** For the first time, we describe heterozygous *RHOT1* mutations in two PD patients (het c.815G>A; het c.1348C>T) and identified mitochondrial phenotypes with reduced mitochondrial mass in patient fibroblasts. Both mutations led to decreased endoplasmic reticulum-mitochondrial contact sites and calcium dyshomeostasis. As a consequence, energy metabolism was impaired, which in turn caused increased mitophagy.

***Innovation and Conclusion:*** Our study provides functional evidence that *ROTH1* is a genetic risk factor for PD, further implicating Miro1 in calcium homeostasis and mitochondrial quality control.

Innovation Miro1 is a crucial player in different pathways involved in the pathogenesis of Parkinson's disease (PD), for example, functional impaired of Miro1 was previously described as a shared feature of defective mitophagy in cellular models of monogenic and sporadic PD. Although *RHOT1* was assumed to be a potential risk factor in PD over several years, no study could show pathogenic mutations. Our results provide first genetic and functional evidence linking disease-associated variants in *RHOT1* with mitochondrial dysfunction in PD, including impaired endoplasmic reticulum-mitochondrial tethering and calcium homeostasis. Thus, our study moves Miro1 more into the focus of potential therapeutic strategies for PD.

## Introduction

The mitochondrial Rho GTPase Miro1 has primarily been studied with respect to its function as an adaptor protein for mitochondrial transport (48, 49), yet far less is known about the involvement of Miro1 in other processes crucial for maintaining mitochondrial homeostasis, such as mitochondrial calcium handling (9), mitochondrial quality control (49, 50), and overall mitochondrial homeostasis (46). Miro1 contains an N-terminal GTPase domain, followed by two calcium-binding EF-hand domains, a second C-terminal GTPase domain, and the C-terminal transmembrane domain. The GTPase domains are involved in the control of mitochondrial movement (30) and in the regulation of mitochondrial calcium uptake (37) *via* the mitochondrial calcium uniporter (MCU) (9). The calcium binding motifs of Miro1 are suggested to ensure the proper spatial arrangement of mitochondrial networks (31, 42, 48) as well as mitochondrial calcium uptake (9).

An initial link between Miro1 and Parkinson's disease (PD) arose from the identification of Miro1 as a target of the PD-associated kinase PINK1 (PARK6) in a mitochondrial quality control pathway (50). Mitochondrial arrest is an important initial step required to isolate dysfunctional mitochondria and to prevent their fusion with healthy mitochondria. As a consequence, immobile fragmented mitochondria are primed for autophagosomal uptake and lysosomal degradation (50). Moreover, in monogenic and sporadic PD, an impairment of Miro1 degradation and mitochondrial dynamics was identified as a central component in neurodegeneration (16).

A recent study in embryonic fibroblasts from Miro1-mutant mice provided evidence for a link between the calcium-sensing function of Miro1 and mitochondrial shape transition (MiST), which is a crucial prerequisite for subsequent mitophagy (31).

Here, we report the identification of mutations in *RHOT1*, the gene encoding Miro1, in PD patients and describe their pathogenic role in the maintenance of endoplasmic reticulum (ER)–mitochondria contact sites, cellular calcium homeostasis, and energy metabolism. Our phenotypic characterization in patient-derived cells points at the multifaceted roles of Miro1 at the outer mitochondrial membrane and highlights the significance of the protein for calcium homeostasis and mitochondrial impairment related to the pathogenesis of PD.

## Results

### Identification of R272Q and R450C *RHOT1* variants in PD patients

**FIG. 1.**
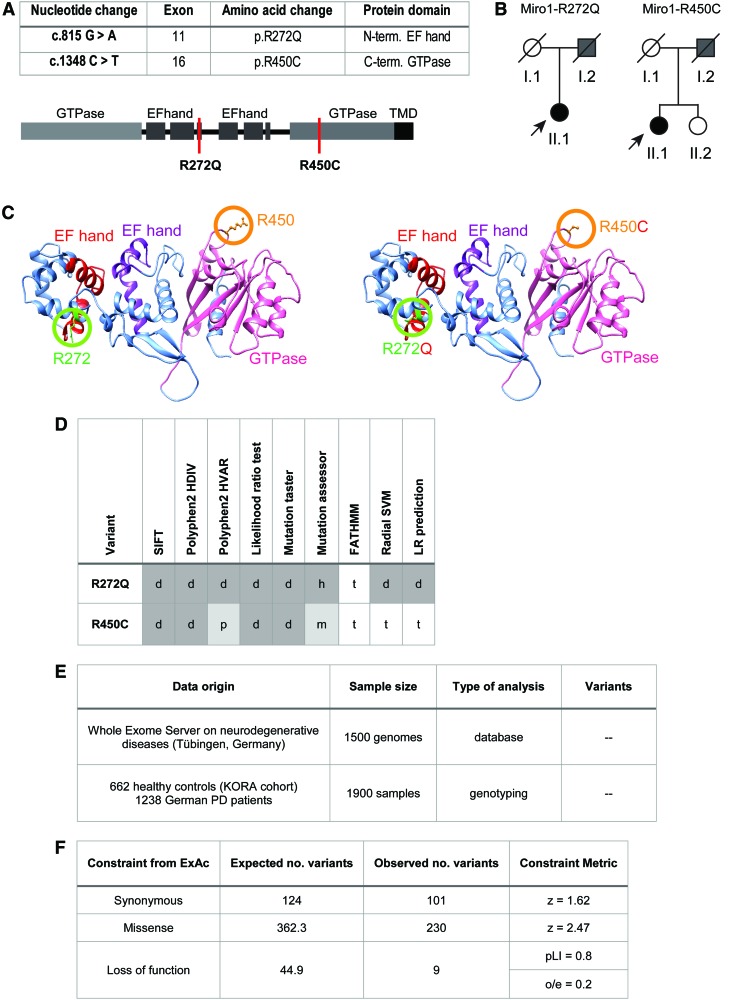
**Identification of R272Q and R450C *RHOT1* variants in PD patients. (A)** Table of point mutations identified in *RHOT1* in two PD patients and location of both mutations within the protein structure of Miro1. Miro1 consists of an N-terminal GTPase domain, followed by two EF-hand domains, a C-terminal GTPase domain, and a TMD. **(B)** Pedigree of PD patients with heterozygous point mutations in *RHOT1*. Individuals displaying motor symptoms are highlighted in *gray*, *arrows* pointing to PD patients of whom fibroblasts were obtained for the present study. **(C)** *Left panel*: homology model of WT human Miro1, covering the 3D protein structure of the both EF-hand domains and the C-terminal GTPase domain. The WT amino acids R272 and R450 are highlighted in *circles*. *Right panel*: homology model of mutant Miro1 with both mutant amino acids R272Q and R450C highlighted in *circles*. **(D)** *In silico* prediction of pathogenic effects of both Miro1 variants. d/h, deleterious/highly functional; p/m, probably damaging/medium functional; t, tolerant. **(E)** Genotyping and data mining of a set of PD and control databases to identify additional carriers of the *RHOT1* mutations. The whole-exome server on neurodegenerative disease, containing 1500 genomes, was searched for additional carriers of the R272Q and R450C variants. Additionally, 1900 samples (662 control individuals from the KORA cohort and 1238 German PD patients) (51) were genotyped to confirm our previous findings. No additional carrier of the R272Q or R450C variants was identified. **(F)** Burden analysis of variants in *RHOT1* from the gnomAD database. LoF variants were defined as single nucleotide exchanges causing nonsense splice acceptor or splice donor variants. Z-score = calculated from expected variant counts divided by observed variant counts. pLI scores close to 1 indicate a high intolerance to LoF variants. 3D, three-dimensional; LoF, loss-of-function; PD, Parkinson's disease; pLI, probability of being LoF intolerant; TMD, transmembrane domain; WT, wild type. Color images are available online.

Since several studies suggested that variants in *RHOT1* may confer risk to develop PD (2, 16, 49), we performed a comprehensive genetic screening for mutations in *RHOT1* in PD patients. In a German cohort of 752 PD patients and a total of 374 healthy controls, we identified 2 female patients carrying a heterozygous mutation c.815G>A or c.1348C>T in *RHOT1* (NM_001033568), leading to the amino acid exchanges R272Q and R450C, respectively (Fig. 1A). The amino acid R272 is positioned within the ligand mimic motif of the first EF-hand domain (20) and the residue R450 lies within the C-terminal GTPase domain (Fig. 1A). According to the homology model of the human Miro1 protein, the affected amino acids are exposed to the cytosol on the protein surface (Fig. 1C). Different *in silico* prediction methods revealed a high likelihood for both mutations to have pathogenic effects (Fig. 1D). Medical records of the fathers of both index patients revealed an unclassified tremor (Fig. 1B). Due to the typical late onset and family history for motor symptoms, both patients were also tested for GBA and LRRK2 mutations. This analysis excluded the GBA N370S and L444P and the LRRK2 G2019S and I2020T mutations by Sanger sequencing in both patients.

To identify additional carriers of the herein described *RHOT1* variants, we next searched a whole-exome database on neurodegenerative diseases containing 1500 patient genomes. This approach revealed no additional carrier of R272Q or R450C mutations. Additional genotyping of 1238 German PD patients (51) and 662 healthy controls (KORA cohort) provided no further carriers of the R272Q or R450C mutation (Fig. 1E).

Using the gnomAD browser to assess the genetic burden of *RHOT1* mutations in the general population, we found a lower amount of missense mutations than expected (missense z-score = 2.47) and a constraint for loss-of-function (LoF) mutations (observed/expected [o/e] score = 0.2; probability of being LoF intolerant (pLI) score = 0.8; Fig. 1F), which further supports a potential pathogenic role of damaging mutations in *RHOT1*.

In the gnomAD database, we found no additional carrier of the R272Q variant, but three carriers of the R450C variant (allele frequency 1.06e-5). Two of these individuals are of European ancestry and the third individual of East Asian origin. All carriers of the R450C variant are heterozygous and their age was between 30 and 35 years. As our patients carrying Miro1 mutations presented with typical late-onset PD, it cannot be excluded that these individuals will develop PD later in life.

From these results, we conclude that damaging mutations in *RHOT1* are a rare event and suggest that mutations may contribute to the development of PD.

### Reduced calcium buffering capacity in Miro1-mutant fibroblasts

**FIG. 2.**
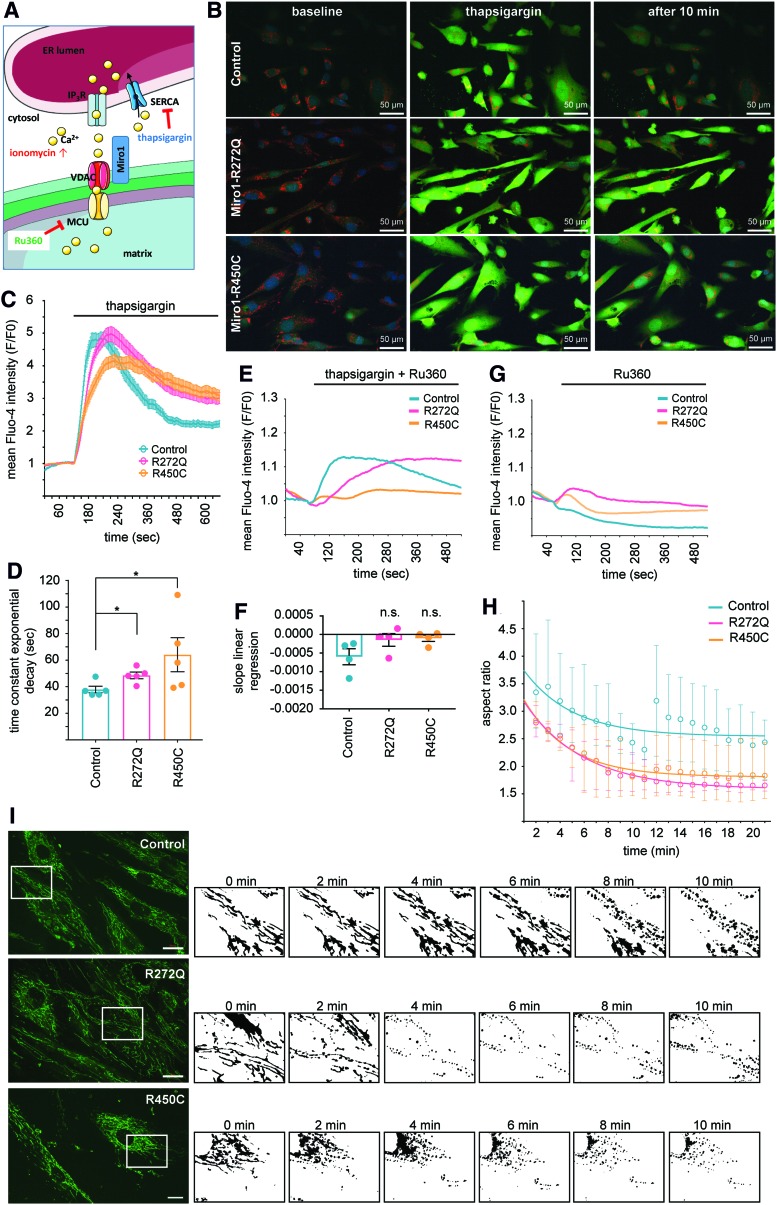
**Reduced calcium buffering capacity in Miro1-mutant fibroblasts. (A)** Overview of treatments with thapsigargin, ionomycin, and Ru360 for calcium imaging. Thapsigargin inhibits calcium uptake of the ER by the SERCA pumps, which leads to increase of cytosolic calcium levels by depletion of ER calcium stores. Ionomycin increases the levels of cytosolic calcium by activation of G-protein-coupled receptors in the plasma membrane. Ru360 is an inhibitor of the MCU. **(B)** Immortalized fibroblasts were loaded with Fluo4-AM (*green*) for live cell imaging. Cells were imaged under baseline condition for 2 min. After addition of 1 μ*M* thapsigargin, imaging was continued for 10 min with a 2 s interval. Images were acquired using a 25 × objective; scale bars indicate 50 μm. **(C)** Quantification of calcium levels upon thapsigargin treatment from **(B)**. Mean intensity of Fluo4-AM signal was indicated as (F/F_0_). Data indicated as mean ± SEM, *n* = 5, with 8–20 cells per cell line per experiment. **(D)** Time constant of exponential decay calculated from calcium response curves of **(C)**. Data indicated as mean ± SEM. Significance calculated using the Mann–Whitney test (*n* = 5). **(E)** Immortalized fibroblasts were stained with Fluo4-AM for live cell imaging. After 1 min of baseline recording, cells were treated with a combination of 10 μ*M* Ru360 and 1 μ*M* thapsigargin, and imaging was continued for 9 min, using a 25 × objective (*n* = 3–4). Data indicated as mean. **(F)** Slope of linear regression of calcium response curves from **(E)**. Data indicated as mean ± SEM. Significance calculated using the Mann–Whitney test (*n* = 3–4). **(G)** Immortalized fibroblasts were stained with Fluo4-AM and treated with 10 μ*M* Ru360 during live cell imaging. Data indicated as mean (*n* = 3). **(H)** Microscopy images from **(I)** were analyzed for aspect ratios of the mitochondrial network over time using the ImageJ (*n* = 3–5). **(I)** Immortalized fibroblasts were stained with MitoTracker^®^ Green FM and treated with 20 μ*M* ionomycin during 20 min of live cell imaging, using a 63 × objective. Scale bars indicate 20 μm. Representative images of the mitochondrial network in control fibroblasts, Miro1-R272Q, and R450C fibroblasts. Mitochondrial masks generated with the ImageJ for the analysis of mitochondrial morphology were displayed at different time points for all cell lines. The *white boxes* in the microscopy images on the *left* highlight the sections depicted on the *right*, showing details of the mitochondrial network at different time points (binary images). Significance for all data calculated by the Mann–Whitney test. *p ≤ 0.05. ER, endoplasmic reticulum; MCU, mitochondrial calcium uniporter; SEM, standard error of the mean. Color images are available online.

One important function of Miro1 is the maintenance of mitochondrial calcium homeostasis (9, 37), which is regulated by the calcium-binding EF-hand motifs and the C-terminal GTPase domain (37). To assess the effects of mutant Miro1 on calcium homeostasis, we used the cytosolic calcium indicator Fluo4-AM. Cells were treated with thapsigargin (an inhibitor of the SERCA pumps) (43), which prevents calcium buffering by the ER (Fig. 2A) and causes an increase of cytosolic calcium by depletion of ER calcium stores (34). In control cells, cytosolic calcium levels decreased within 5 min, whereas in Miro1-R272Q and in Miro1-R450C fibroblasts, the cytosolic calcium content remained increased (Fig. 2B, C).

The alteration of calcium handling was also reflected by significantly increased time constants of exponential decay, calculated from the calcium response curves after treatment with thapsigargin (Fig. 2C), in both Miro1-mutant fibroblast lines compared with controls (Fig. 2D). To distinguish whether the altered calcium profiles in mutant fibroblasts were due to impaired mitochondrial calcium buffering or a reduced calcium release across the plasma membrane, we next treated the cells with Ru360, an inhibitor of the MCU (9, 18, 44), alone or in combination with thapsigargin (Fig. 2E, G). Blocking of the MCU after thapsigargin treatment led to a reduced buffering capacity of cytosolic calcium in mutant and control cells (Fig. 2E).

This observation was confirmed by calculation of the linear regression of the calcium response to concomitant thapsigargin and Ru360 treatment. The slopes of the respective linear regressions were statistically not different between control and mutant fibroblasts (Fig. 2F), indicating a disruption of the calcium buffering capacity when mitochondrial calcium uptake was blocked by Ru360. These results suggest that the reduced capacity of Miro1 mutant fibroblasts to buffer cytosolic calcium is due to impaired mitochondrial buffering.

This hypothesis was further strengthened by data resulting from the analysis of calcium-induced MiST, a mechanism that was recently identified as a prerequisite of mitophagy and that is dependent on the ability of Miro1 to regulate calcium homeostasis (31). Fibroblasts were stained with MitoTracker^®^ Green FM and treated with the calcium ionophore ionomycin to increase cytosolic calcium levels. Independent of their genotype, all cells reacted to the ionomycin exposure with mitochondrial fragmentation as reflected by a decrease in the aspect ratios. However, in both Miro1-mutant fibroblast lines, we observed an increased fragmentation of the mitochondrial network compared with control fibroblasts (Fig. 2H, I). These results suggest that the impaired calcium buffering capacity of Miro1-mutant cells results in prolonged and elevated cytosolic calcium levels, which subsequently cause an increased induction of calcium-mediated mitochondrial fragmentation.

### Decreased ER–mitochondrial contact sites in Miro1-mutant fibroblasts

**FIG. 3.**
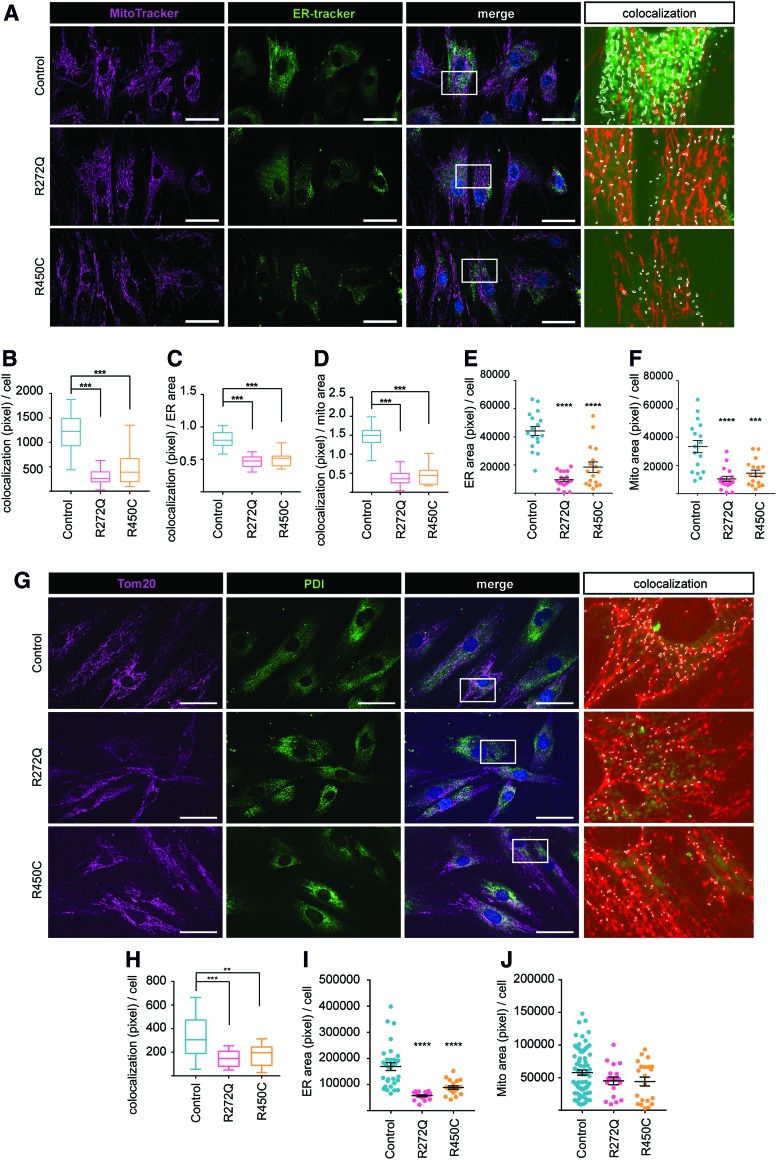
**Decreased ER**–**mitochondrial contact sites in Miro1-mutant fibroblasts. (A)** Native fibroblasts were stained with MitoTracker Deep Red (*magenta*) and ER-Tracker Green. Images were obtained with a 40 × objective (*n* = 3). Scale bars indicate 50 μm. Colocalization of ER and mitochondria was analyzed in the merged-channel images. The *white squares* in the merged images indicate the zoomed regions shown for the colocalization panel; colocalization events are highlighted as *white dots* (see *colocalization panel*). **(B)** Colocalization events of mitochondria and ER pixel from **(F)** were normalized to cell number, **(C)** to ER area, or **(D)** to mitochondrial area. **(E)** ER area (pixel) per cell was analyzed from live cell microscopy data of ER-Tracker. **(F)** Mitochondrial area (pixel) per cell was analyzed from live cell microscopy data of MitoTracker Deep Red. **(G)** Native fibroblasts were fixed and labeled with antibodies against Tom20 (*magenta*) and PDI (*green*) for subsequent immunofluorescent microscopy analysis of mitochondria and ER colocalization. Colocalization of ER and mitochondria was analyzed in the merged-channel images. The *white squares* indicate the zoomed regions shown in the colocalization panel. Colocalization events are highlighted as *white dots*. Images were obtained with a 40 × objective. **(H)** Colocalization events of mitochondria and ER pixel from **(G)** were normalized per cell (*n* = 3, with approximately 80–90 cells per cell line per experiment). **(I)** ER area (pixel) per cell was analyzed by quantification of the PDI staining. **(J)** Mitochondrial area (pixel) per cell was analyzed by quantification of the Tom20 staining. Significance was calculated by the Kruskal–Wallis test (*n* = 3). All data indicated as mean ± SEM. **p < 0.01; ***p ≤ 0.001; ****p < 0.0001. Color images are available online.

Closely related to its function in cellular calcium homeostasis, Miro1 is also involved in the regulation of ER–mitochondria contact sites (21). In light of the observed impaired mitochondrial calcium buffering in Miro1-mutant fibroblasts, we next studied the colocalization of the ER and mitochondria using MitoTracker Deep Red and ER-Tracker Green (Fig. 3A). We observed a significant reduction of ER–mitochondria contacts in both patient-derived fibroblast lines compared with control lines, when normalized to cell count (Fig. 3B), ER area (Fig. 3C), or mitochondrial area (Fig. 3D).

Intriguingly, we also observed an overall reduction of ER area (Fig. 3E) and mitochondrial area (Fig. 3F) in Miro1-R272Q and R450C fibroblasts. These results were confirmed by immunocytochemistry of ER and mitochondrial marker proteins PDI and Tom20 (Fig. 3G). Subsequent colocalization analysis showed a significant reduction of ER–mitochondria contacts in Miro1-mutant fibroblasts (Fig. 3H), and ER area was likewise significantly reduced (Fig. 3I), whereas mitochondrial area showed a tendency to be reduced in Miro1-mutant fibroblasts compared with controls (Fig. 3J).

Our results point to a reduction of ER–mitochondria contact sites in fibroblasts with mutations in Miro1.

### Mutant Miro1 protein leads to reduction of mitochondrial mass

**FIG. 4.**
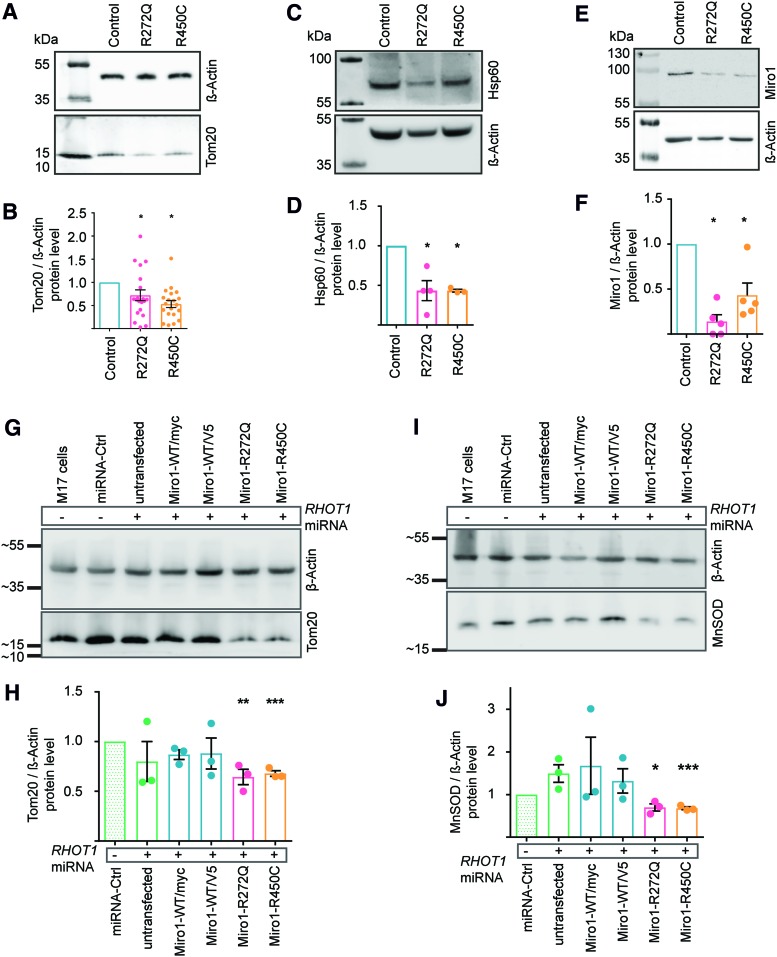
**Mutant Miro1 protein leads to reduction of mitochondrial mass. (A)** Representative Western blot of Tom20 in immortalized fibroblasts. **(B)** Quantification of Tom20 protein level in immortalized fibroblasts from **(A)**. Significance tested with the Wilcoxon test (*n* = 20). **(C)** Representative Western blot image of Hsp60 protein in immortalized fibroblasts. **(D)** Quantification of Hsp60 normalized to β-actin from **(C)**. Significance assessed using the Mann–Whitney test (*n* = 4). **(E)** Representative Western blot image of Miro1 protein in immortalized fibroblasts. **(F)** Quantification of Western blot analysis of Miro1 protein levels from **(E)**. Significance determined using the Wilcoxon test (*n* = 5). **(G)** Representative Western blot image of Tom20 and **(I)** MnSOD proteins in M17 cells with stable knockdown of endogenous *RHOT1* and transiently overexpression of Miro1 variants. (−) Indicates M17 cells without knockdown of endogenous *RHOT1*. (+) Indicates knockdown of endogenous *RHOT1* by stable transfection with the *RHOT1*-targeting miRNA-2471 (*RHOT1* miRNA). M17 cells were transfected with Miro1-WT/myc (in pRK5-myc vector), Miro1-WT/V5, Miro1-R272Q, or Miro1-R450C (in pcDNA3.1/V5-HisA vector). **(H)** Quantification of Tom20 protein levels from **(G)** (*n* = 3). **(J)** Quantification of MnSOD protein levels from **(I)** (*n* = 3). All data indicated as mean ± SEM. *p ≤ 0.05; **p ≤ 0.01; ***p ≤ 0.001. miRNA, microRNA. Color images are available online.

Based on the reduced mitochondrial area observed during our microscopy analysis of ER–mitochondria contact sites (Fig. 3F), we further investigated mitochondrial mass in Miro1-mutant fibroblasts. To this end, we performed Western blotting analyses of different mitochondrial marker proteins. First, we confirmed a reduction of Tom20 in the mutant fibroblasts (Fig. 4A, B). In addition, we quantified the abundance of the mitochondrial matrix marker Hsp60 (8, 35). Indeed, Hsp60 protein levels were found to be significantly reduced in mutant compared with control fibroblasts (Fig. 4C, D). Finally, we investigated the protein levels of Miro1 itself and also confirmed a reduction in fibroblasts expressing mutant Miro1 (Fig. 4E, F).

As both the Miro1-R272Q and the Miro1-R450C mutant fibroblast lines were heterozygous, these results raised the question whether the observed mitochondrial phenotypes are due to a gain-of-function effect or due to the loss of Miro1 function. To answer this question, we generated an M17 cell model with stable knockdown of endogenous *RHOT1* and transient overexpression of the PD-associated Miro1 variants (Supplementary Fig. S1). Western blot analyses revealed that knockdown of *RHOT1* alone (untransfected M17 cells+microRNA [miRNA]) had no effect on protein levels of Tom20 or the mitochondrial matrix marker MnSOD (8, 35), whereas overexpression of Miro1-R272Q or Miro1-R450C, but not wild type (WT)-Miro1, led to a significant reduction of Tom20 (Fig. 4G, H) and MnSOD (Fig. 4I, J), respectively. From these results, we concluded that the R272Q and R450C mutations in Miro1 cause a toxic gain of function.

### Mitochondrial turnover is increased in Miro1-mutant fibroblasts

**FIG. 5.**
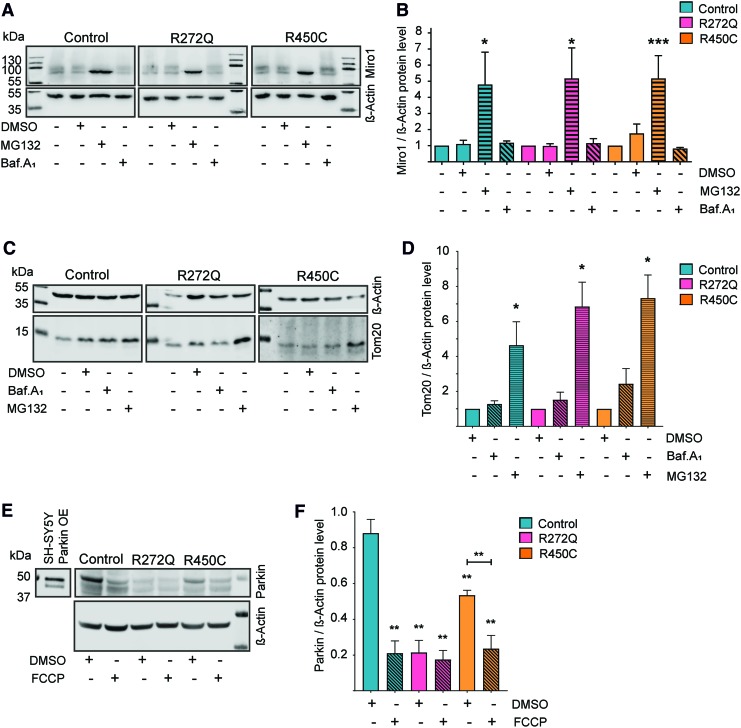
**Mitochondrial turnover is increased in Miro1-mutant fibroblasts. (A)** Representative Western blot image of Miro1 in immortalized fibroblasts treated with 10 μ*M* MG132 for 24 h or with 10 n*M* bafilomycinA_1_ for 48 h. **(B)** Quantification of Miro1 protein level from Western blots displayed in **(A)**. Significance assessed with the Wilcoxon test (*n* = 3–7). **(C)** Western blot image for Tom20 protein in immortalized fibroblasts treated with 10 n*M* bafilomycinA_1_ for 48 h or with 10 μ*M* MG132 for 24 h, respectively. **(D)** Quantification of Tom20 protein levels from Western blot analysis shown in **(C)**. Significance assessed using the Wilcoxon test (*n* = 5). **(E)** Representative Western blot image of Parkin protein. *Left panel* shows Parkin bands in SH-SY5Y cells overexpressing Parkin. *Right panel* shows endogenous Parkin in immortalized fibroblasts treated with 10 μ*M* FCCP for 14 h. **(F)** Quantification of Parkin protein levels normalized to β-actin from **(E)**. Significance calculated by the Mann–Whitney test (*n* = 5). All data indicated as mean ± SEM. *p ≤ 0.05; **p ≤ 0.01; ***p ≤ 0.001. FCCP, carbonyl cyanide-4-(trifluoromethoxy)phenylhydrazone. Color images are available online.

Previous studies reported that the PINK1/Parkin-driven proteasomal degradation of Miro1 is part of the initial step of mitophagy (4, 28, 38, 49, 50). Given the observed reduced levels of Miro1 protein and mitochondrial mass in Miro1-mutant cells (Fig. 4), we were interested to investigate the underlying degradation pathways. Patient-derived fibroblasts were therefore treated with the proteasomal inhibitor MG132 and bafilomycinA_1_, an inhibitor of the lysosomal degradation pathway. Western blot analysis showed an increase of Miro1 protein levels (Fig. 5A, B) as well as Tom20 protein levels (Fig. 5C, D) after MG132 treatment, but not after bafilomycinA_1_ treatment, suggesting that both Miro1 and Tom20 are predominantly degraded by the proteasome. This result is in line with the literature, indicating that Miro1 and Tom20 are targeted by PINK1/Parkin for proteasomal degradation during mitophagy (4, 8, 28, 35, 38, 52). To further test this hypothesis, we assessed Parkin levels in patient-derived fibroblasts. Carbonyl cyanide-4-(trifluoromethoxy)phenylhydrazone treatment for 14 h significantly decreased Parkin levels in control fibroblasts, suggesting an activation of mitophagy (Fig. 5E, F). By contrast, Parkin levels were already found to be significantly reduced at baseline conditions in both Miro1-mutant fibroblast lines (Fig. 5F). These results, together with the reduction of mitochondrial mass (Fig. 4), support that mitochondria undergo increased clearance in Miro1-mutant fibroblasts under basal conditions.

### LC3-dependent autophagy is affected in Miro1-mutant fibroblasts

**FIG. 6.**
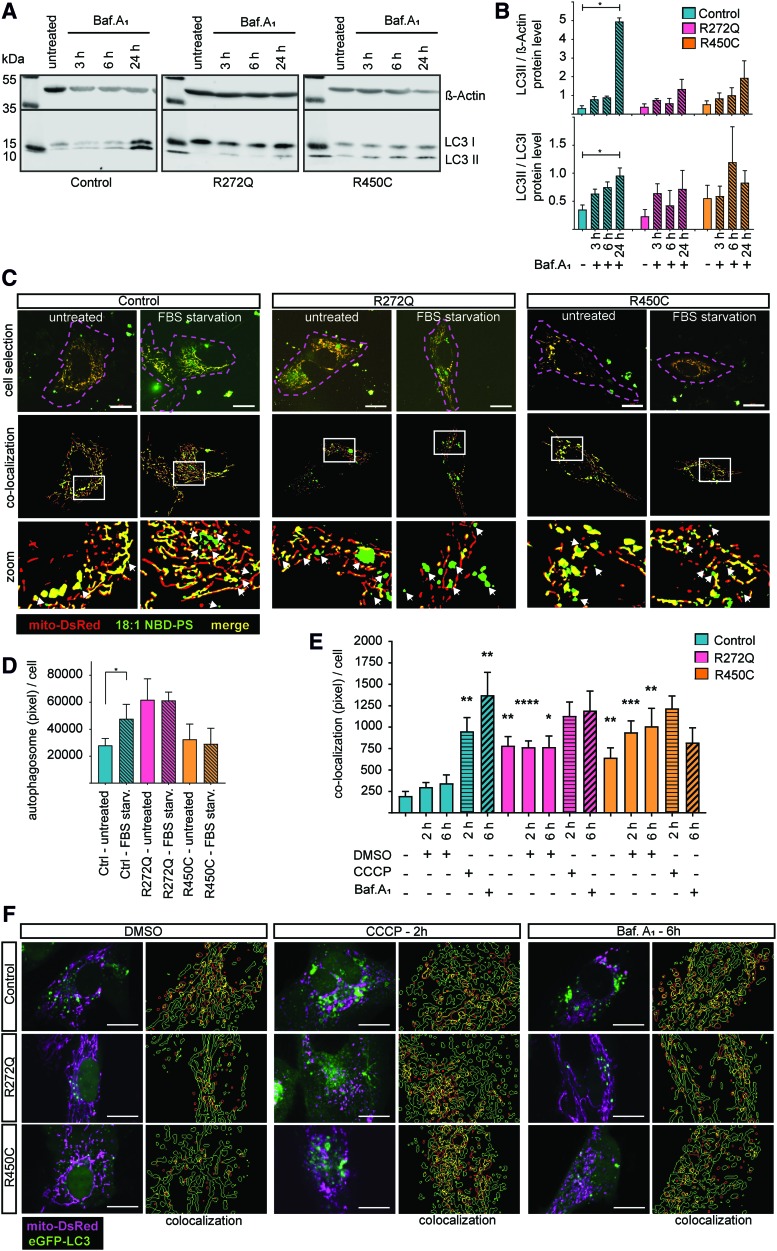
**LC3-dependent autophagy is affected in Miro1-mutant fibroblasts. (A)** Representative Western blot image of immortalized fibroblasts treated with 10 n*M* bafilomycinA_1_ for 3, 6, or 24 h. **(B)** Densitometry analysis of Western blot for LC3-II normalized to β-actin (*upper graph*) and the ratio of LC3-II/LC3-I protein levels (*lower graph*) from **(A)**. Significance calculated by the Friedman test (*n* = 3). **(C)** Immortalized fibroblasts were transfected with mito-DsRed and labeled with 18:1 NBD-PS. Then, cells were starved in medium without FBS for 2 h for subsequent live cell imaging, using a 63 × objective. Cell selection shows microscopy images with mito-DsRed-labeled mitochondria (*red*) and 18:1 NBD-PS (*green*). Cells were manually selected (*dashed pink outline*) to ensure that only 18:1 NBD-PS-labeled autophagosomes in the cytosol were analyzed. The colocalization panel shows the selected cells from the data analysis. *White squares* indicate the regions, which were shown in the *zoom panel*. Autophagosomes were identified as *green* particles, which are not colocalizing with the mito-DsRed signal (*white arrows*). **(D)** Quantification of autophagosome formation from images shown in **(C)**. Significance calculated by the Wilcoxon test (*n* = 3). **(E)** Colocalization of mitochondria and LC3 pixel from **(F)** were quantified and normalized to cell number. Significance assessed using the Mann–Whitney test (*n* = 3, with ∼30 cells per cell line). **(F)** Immortalized fibroblasts were transfected with mito-DsRed and eGFP-LC3. Twenty-four hours after transfection, cells were treated with 25 μ*M* CCCP or with 10 n*M* bafilomycinA_1_ for 2 or 6 h, respectively. The *left panels* show the microscopy images obtained with a 40 × objective. The *right panels* show the colocalization analysis. The outlines of analyzed mitochondria are indicated in *green*, whereas the outlines of analyzed LC3 puncta are indicated in *red*. The colocalization regions of both organelles are indicated in *yellow*. Scale bars indicate 20 μm. All data indicated as mean ± SEM. *p ≤ 0.05; **p ≤ 0.01; ***p ≤ 0.001; ****p < 0.0001. FBS, fetal bovine serum; PS, phosphatidylserine. Color images are available online.

Since we observed reduced mitochondrial mass (Fig. 4) and activation of mitochondrial clearance (Fig. 5), we next analyzed autophagy in immortalized Miro1-mutant and control fibroblasts. To assess autophagic flux, we treated cells with bafilomycinA_1_. Western blot analysis revealed that LC3-II protein levels normalized to β-actin (19), as well as the ratio of LC3-II to LC3-I protein levels increased significantly in control fibroblasts, but not in the Miro1-mutant fibroblast lines (Fig. 6A, B), indicating a reduced capacity to enhance the autophagic flux.

**FIG. 7.**
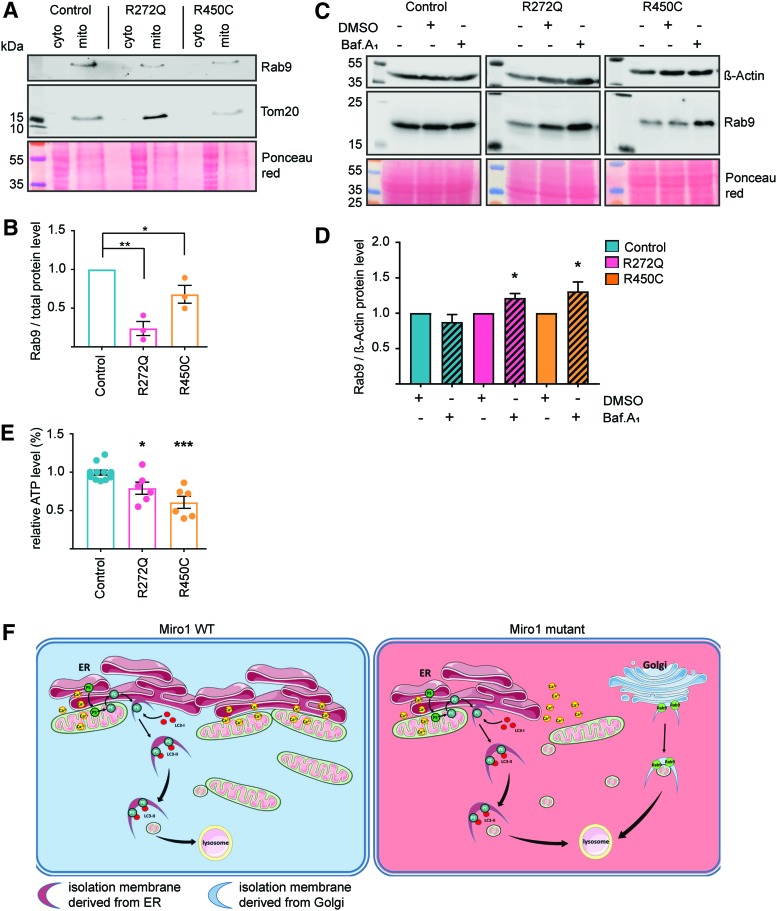
**Mutations in Miro1 lead to increased LC3-independent autophagy and impaired energy metabolism. (A)** Crude mitochondrial fractions were obtained from immortalized fibroblasts. Rab9 levels were analyzed by Western blot. **(B)** Quantification of Rab9 protein levels in mitochondrial fractions from **(A)** (*n* = 3). **(C)** Representative Western blot image of Rab9 in whole cell lysates of immortalized fibroblasts treated with 10 n*M* bafilomycinA_1_ for 4 h. **(D)** Quantification of Rab9 protein levels from **(C)**. Significance determined using the Wilcoxon test (*n* = 6–11). **(E)** Measurement of steady-state ATP level under baseline conditions in immortalized fibroblasts. Significance assessed with the Mann–Whitney test (*n* = 6). **(F)** Schematic overview of the mechanism of impaired ER–mitochondria contact sites and increased mitophagy in Miro1 mutant background. *p < 0.05; **p < 0.01; ***p < 0.001. Color images are available online.

To further characterize the autophagy pathway and based on our findings of reduced ER–mitochondrial contact sites in cells expressing mutant Miro1, we stained fibroblasts with fluorescently labeled phosphatidylserine (PS) (18:1 NBD-PS). PS is transferred at ER–mitochondria contact sites from the ER to mitochondria, where it is converted to phosphatidylethanolamine (PE). PE is then transferred through the ER–mitochondria contact sites back to the ER and contributes together with the cytosolic LC3-I to create autophagosomes (Fig. 7F). In this assay, the formation of autophagosomes is reflected by translocation of the 18:1 NBD-PS fluorescence signal from mitochondria to the cytosol (14, 31) (Fig. 6C). Fetal bovine serum (FBS) starvation for 2 h induced autophagosome formation in control fibroblasts, whereas Miro1-mutant fibroblasts showed no increased autophagosome formation compared with the untreated condition (Fig. 6D). To specifically analyze mitophagy, fibroblasts were transfected with mito-DsRed and eGFP-LC3 and subsequently treated with carbonyl cyanide 3-chlorophenylhydrazone (CCCP) to induce mitophagy or with bafilomycinA_1_ to inhibit autophagy. Mitophagy was subsequently assessed by quantification of mitochondria colocalizing with LC3 puncta, which allowed us to analyze only those autophagosomes that are involved in mitochondrial degradation (Fig. 6E, F) (19). Under CCCP or bafilomycinA_1_ treatment, the colocalization of mito-DsRed-labeled mitochondria and eGFP-LC3 puncta significantly increased in control fibroblasts, whereas no significant increase could be observed in Miro1-mutant fibroblasts, compared with baseline conditions (Fig. 6E). Our results support the idea of already elevated mitophagy rates in the R272Q and R450C fibroblasts at baseline, as indicated by a higher frequency of mitochondria-LC3 colocalizing events in the untreated mutant cells compared with untreated control cells (Fig. 6E). These results suggest that, while the total autophagosome formation under baseline conditions is not affected in Miro1-muant cells compared with controls, the capacity to further upregulate autophagosome formation is inhibited in those cells (Fig. 6D). Furthermore, the created autophagosomes are involved in increase mitochondrial turnover, which likewise lacks the capacity for further upregulation under stress conditions (Fig. 6E).

### Mutations in Miro1 lead to increased LC3-independent autophagy and impaired energy metabolism

Our results suggest alterations of LC3-dependent autophagy in Miro1-mutant fibroblasts (Fig. 6), significantly reduced mitochondrial mass (Figs. 3 and 4) and increased mitochondrial clearance (Fig. 5). Therefore, we also investigated an alternative LC3-independent clearance pathway, where autophagosomes are derived from the Golgi apparatus and their formation is mediated by Rab9 (33). When analyzing protein levels of Rab9 in cytosolic and mitochondrial fractions by Western blotting, we found that Rab9 levels were significantly reduced in mitochondrial fractions of Miro1-mutant fibroblasts compared with control fibroblasts (Fig. 7A, B). To test whether these results were linked to autophagy, we treated fibroblasts with bafilomycinA_1_. This experiment showed an increase in Rab9 levels after bafilomycinA_1_ treatment in Miro1-mutant fibroblasts, but not in control cells (Fig. 7C, D), suggesting a higher lysosomal turnover of Rab9. From these results, we concluded that mitochondrial degradation in Miro1-mutant fibroblasts is additionally driven by the Atg5/LC3-indipendent macroautophagy pathway.

Finally, since mitophagy and calcium homeostasis are closely linked to mitochondrial energy metabolism, we next sought to investigate the ATP production in our patient and control cultures. Measuring cellular ATP concentrations under baseline conditions showed significantly reduced steady-state levels in both mutant fibroblast lines compared with control cells (Fig. 7E).

## Discussion

Increasing evidence supports a contribution of impaired energy metabolism and mitochondrial dynamics to the pathogenesis of monogenic and sporadic PD, that is, for at least seven established PD genes (PINK1, Parkin, DJ-1, LRRK2, ATP13A2, SNCA, and VPS35), a role in mitochondrial homeostasis and clearance has been described (24). Within this context, the encoded proteins interact with other targets, which are potentially relevant to the pathogenesis of PD. One of these targets is Miro1, which has been shown to directly interact with PINK1 and Parkin (4). Moreover, recent studies suggest a function of Miro1 together with LRRK2 in the regulation of damage-induced mitochondrial arrest (16). The pathological relevance of Miro1 was supported by the lethality of newborn homozygous *RHOT1*-knockout mice (32) and larvae of *dMiro*-knockout flies (13).

Here, we describe for the first time phenotypes in fibroblasts derived from PD patients carrying mutations in *RHOT1*. These mutations lead to a decrease in ER–mitochondria contact sites, which consequently caused (i) impaired cellular calcium homeostasis and (ii) increased calcium-induced mitochondrial fragmentation. These phenotypes ultimately (iii) induce mitochondrial clearance. However, the LC3-dependent formation of autophagosomes lacks the capacity to further promote mitophagy under stress conditions. Mitophagy is therefore additionally driven by LC3-independent autophagy *via* Rab9, resulting in (iv) reduced mitochondrial mass in Miro1-mutant cells (see overview Fig. 7F).

The herein described Miro1-muant fibroblast lines displayed strikingly similar phenotypes, although the R272Q and R450C mutations are located in different domains of the protein, that is, the EF-hand and the C-terminal GTPase domain. Both domains have been shown to form a unique side-by-side three-dimensional structure that facilitates their close interplay (20), which is crucial for the calcium-sensing ability of Miro1 (9, 20, 21, 37, 42). In light of these previous reports, we conclude from our data that both mutations in Miro1 disrupt the proteins calcium-sensing function.

The importance of cellular calcium regulation at ER–mitochondrial contact sites for the function and survival of neuronal cells was recently highlighted by studies in *drosophila*. Lee *et al.* identified Miro1 as regulator of calcium transporters at the ER–mitochondrial contact sites, a function that was independent of Miro1's role in mitochondrial transport. Inactive Miro1 was found to cause mitochondrial calcium depletion and metabolic dysfunction, leading to impaired neuronal stem cell development in flies (26).

ER–mitochondrial contact sites not only regulate cytosolic and mitochondrial calcium homeostasis but also autophagy. Within this mechanism, Miro1 acts as receptor at the outer mitochondrial membrane to sense cytosolic calcium levels. Upon increase of calcium levels, Miro1 mediates Drp1/Fis1-independent mitochondrial fragmentation, also called MiST, and subsequent mitophagy. Disruption of the calcium binding ability of Miro1 leads to impaired regulation of calcium homeostasis and mitophagy (31). Indeed, this is in line with our findings of impaired cellular calcium homeostasis and increased calcium-induced mitochondrial fragmentation in Miro1-mutant fibroblasts.

Calcium dyshomeostasis is commonly observed in neurological diseases, and recently, the regulation of calcium homeostasis and autophagy at ER–mitochondrial contact sites came into focus in the context of PD. *Drosophila* expressing a PINK1 LoF mutation or the PD-associated mutation G2019S in LRRK2 displayed impaired calcium homeostasis at ER–mitochondrial contact sites, which resulted in mitochondrial dysfunction and loss of dopaminergic neurons, and Miro1 was identified as key player in this process (25). Also, Parkin was found to be involved in the regulation of ER–mitochondria contact sites. Mirroring the phenotypes found in Miro1-mutant fibroblasts in this study, Calì *et al.* showed that knockdown of Parkin in SH-SY5Y cells results in mitochondrial fragmentation, alterations of mitochondrial calcium transients, and a reduction of ER–mitochondria contact sites. They concluded that, just like Miro1, Parkin is involved in the maintenance of the mitochondrial network integrity *via* the regulation of the ER–mitochondria contact sites and calcium transfer between both organelles (7).

Similar observations were made in S2R+ *drosophila* cells or mouse embryonic fibroblasts with knockdown of Parkin and fibroblasts derived from a PD patient with compound heterozygous mutant Parkin (R275W, exon 3 deletion) (3). The results of this study suggested that Parkin regulates the function of ER–mitochondrial contact sites by ubiquitination of Mfn2 (3). Also, LoF models of Parkin and PINK1 in fly ventral neurons and human induced pluripotent stem cell-derived hypothalamic neurons resulted in alterations of ER–mitochondrial connections and a thereof resulting deregulation of PS transfer, in turn leading to impaired production of neuropeptide-containing vesicles. The resulting changes of neuropeptidergic neurotransmission caused impaired sleep patterns in the PD fly model (45).

It is worth noting that some studies found an increase of ER–mitochondria contact sites, where others found a decrease, caused by impaired PINK1, Parkin, or Miro1 function. These differences seem to be cell type-specific, as knockdown of Parkin in nonneuronal mitotic cells leads to a reduction of ER–mitochondrial connections (3, 7), but knockdown of PINK1, Parkin, or Miro1 in neurons was associated with increased contact sites (25, 45).

In conclusion, our results support an important role of Miro1 in cellular calcium homeostasis and autophagy, highlighting *RHOT1* as candidate gene in the pathogenesis of neurodegenerative disorders such as PD.

## Materials and Methods

### Screening for PD patients with mutations in *RHOT1*

DNA was obtained from 752 German PD patients (average age of onset of 59.4 ± 13.2 years, average age of sample collection of 65.7 ± 10.2 years) and 282 age-matched healthy control individuals (average age 72 ± 4.4 years) from the MEMO study (5). Informed consent was obtained from patients and approved by the ethics committee of the Medical faculty and the University Hospital Tübingen, Germany. Ninety-two samples of healthy individuals from the TREND study (15) served as additional control cohort. Polymerase chain reaction amplification of exons 2–21 of *RHOT1* (Ensembl.org: RHOT1-002, ENST00000358365) from whole-blood DNA samples was performed using the primers listed in Supplementary Table S1 (Metabion, Germany). SYBR Green on the LightCycler 480 High Resolution Master (Roche) and subsequent high-resolution melting analysis were used for mutation screening. Potential mutation sites were validated by Sanger sequencing.

Genotyping of additional cohorts for the herein described *RHOT1* variants was performed by the central genotyping core at the Institute of Human Genetics, Helmholtz Zentrum München, Neuherberg, Germany, using a matrix-assisted laser desorption/ionization time-of-flight mass spectrometry on a MassARRAY System (Agena Bioscience, San Diego, CA). We included 662 age-matched control individuals from the KORA cohort (KORA, Cooperative Research in the Region of Augsburg, Germany) and 1238 PD patients of German origin (51). The genotyping core was blinded to case–control status. Cleaned extension products were analyzed by a mass spectrometer (Bruker Daltonik) and peaks identified using the MassARRAY Typer 4.0.2.5 software (Agena Bioscience). The average call rate of the variants was 97%.

### *In silico* prediction of pathogenic effects of *RHOT1* mutations

ANNOVAR (47) was used to annotate the *RHOT1* mutations using the dbNSFP database version 3.0 (29). The following prediction tools were used: SIFT (23), PolyPhen2 (1), Mutation Taster (39), Mutation assessor (36), FATHMM (40), LRT (likelihood ratio test) (10), radial SVM, and LR pred (11).

### Three-dimensional modeling of human Miro1

A homology model for human Miro1 was derived from the crystal structure of the drosophila ortholog (PDB: 4COJ) using the I-TASSER software with default parameters (53). To visualize the mutations in the homology model, the WT residues were replaced by the rotamers with the highest probability using the “Rotamers” tool in the software Chimera (41), and an energy minimization was applied to these residues with standard settings.

### Genetic burden analysis for *RHOT1*

We used the gnomAD browser to analyze the mutational burden for *RHOT1* (27). Z-scores for missense and synonymous variants were calculated from numbers of observed over expected variants and positive z-scores indicate that there are fewer variants than statistically expected. The o/e score and the pLI score indicate the tolerance of a gene against LoF variants. Low o/e values indicate that the gene is under stronger selection. The closer to 1, the less tolerant the gene is against LoF variants and genes with pLI ≥0.9 are considered to be extremely intolerant to LoF variants (27).

### Immortalization and culture of fibroblasts

Skin biopsies were obtained from two female PD patients at the age of 78 years (Miro1-R272Q) or 54 years (Miro1-R450C). Control fibroblasts were gender- and age-matched to patient-derived fibroblasts and obtained from the Neuro-Biobank of the University of Tübingen, Germany. Informed written consent of all individuals was obtained at the University Hospital Tübingen, Germany. Fibroblasts were immortalized with a pLenti-III-SV40 construct (Cat. No. G203; Applied Biological Materials, Inc., Richmond, Canada). All fibroblasts were grown in Dulbecco's modified Eagle's medium (DMEM)+/+ (containing 4.5 g/L d-glucose, 15% FBS, 1% Pen/Strep; Thermo Fisher Scientific, Braunschweig, Germany). Cells were tested for *Mycoplasma* contamination on a monthly base using the Plasmo Test™ Detection Kit (InvivoGen).

### Generation of M17 model with knockdown of endogenous *RHOT1* and overexpression of Miro1

The human neuroblastoma cell line M17 was grown in DMEM/F12+/+ medium (DMEM/Ham's-F12 + 15% FBS +1% l-glutamine +1% nonessential amino acids +1% Pen/Strep). Cells were split using Trypsin-EDTA (0.05%) and phenol red (Thermo Fisher Scientific). We introduced a stable knockdown of endogenous *RHOT1* employing the BLOCK-iT Inducible Pol II miR RNAi Expression Vector Kit (Invitrogen GmbH, Karlsruhe, Germany) according to the manufacturer's protocol. We designed the following single-stranded nucleotide oligomers, targeting different regions of *RHOT1*: miRNA-524 (top strand: 5′ TGC TGT TTA TGA GAG GAA TCC ATC GAG TTT TGG CCA CTG ACT GAC TCG ATG GAC CTC TCA TAA A 3′; bottom strand: 5′ CCT GTT TAT GAG AGG TCC ATC GAG TCA GTC AGT GGC CAA AAC TCG ATG GAT TCC TCT CAT AAA C 3′); miRNA-1335 (top strand: 5′ TGC TGT AAA TAA GTC GTG AGC GTC CAG TTT TGG CCA CTG ACT GAC TGG ACG CTC GAC TTA TTT A 3′; bottom strand: 5′ CCT GTA AAT AAG TCG AGC GTC CAG TCA GTC AGT GGC CAA AAC TGG ACG CTC ACG ACT TAT TTA C 3′); or miRNA-2471 (top strand: 5′ TGC TGT ATG CTA GCC AAT ACT GCA GTG TTT TGG CCA CTG ACT GAC ACT GCA GTT GGC TAG CAT A 3′; bottom strand: 5′ CCT GTA TGC TAG CCA ACT GCA GTG TCA GTC AGT GGC CAA AAC ACT GCA GTA TTG GCT AGC ATA C 3′). The oligomers were cloned into the pcDNA6.2-GW/EmGFP-miR vector provided by the kit and transfected into M17 cells. Cells were subsequently selected and continuously maintained with 6 μg/mL Blasticidin S HCl (Invitrogen GmbH). Knockdown of endogenous *RHOT1* was verified by Western blot analysis (Supplementary Fig. S1A). For our experiments, we choose the miRNA-2471, which showed the most efficient knockdown. As miRNA-2471 was designed to target the 5′ UTR (untranslated region) of *RHOT1* instead of the open reading frame, we were able to overexpress recombinant Miro1, using *RHOT1* variants cloned into pcDNA3.1/V5-HisA (Invitrogen). M17 cells were transiently transfected with these constructs using *Trans*IT^®^-2020 transfection reagent (MIR 5400; Mirus Bio, LLC, Madison, WI). Overexpression was also verified by Western blot analysis (Supplementary Fig. S1B, C).

### Overexpression of Parkin in SH-SY5Y cells

Neuroblastoma (SH-SY5Y) cells stably overexpressing Parkin were generated using lentiviral particles. To produce Parkin-expressing lentiviral particles, a cassette consisting of the open reading frame of the Parkin gene (NM_004562.3), the IRES sequence, and the Puromycin resistance gene were cloned into pLenti4/V5-DEST (ThermoFisher) plasmid (pLenti4-TH-IRES-Puromycin). Next, 293FT cells were cotransfected with pLenti-Parkin-IRES-Puromycin and the ViraPowerTM Packaging Mix (ThermoFisher) to generate a lentiviral stock. SH-SY5Y cells were transduced using lentiviral particles for 48 h and subsequently selected by using 2 μg/mL Puromycin (ThermoFisher) for 48 h.

### Sodium dodecyl sulfate polyacrylamide gel electrophoresis and Western blot analysis

Fibroblasts were lysed in RIPA buffer containing 1 × complete protease inhibitor (Roche). Western blot analysis was performed with antibodies against Miro1 (WH0055288M1; Sigma–Aldrich, Munich, Germany), LC3-I/II (2775; Cell Signaling), Hsp60 (4870; Cell Signaling), Tom20 (sc-17764; Santa Cruz Biotechnologies), Rab9 (sc-74482; Santa Cruz Biotechnologies), MnSOD (ab13533; Abcam), anti-V5 (R960-25; Novex, R96125; Sigma–Aldrich), and β-actin (MA1-744; Thermo Scientific). Mitochondrial fractionation was performed as described previously (12).

### Live cell imaging

Fibroblasts were seeded into Nunc™ Lab-Tek™ Chamber slides (Thermo Fisher Scientific) for imaging. Live cell imaging was performed with a Live Cell Microscope Axiovert 2000 with spinning disc, plan-apochromat objectives, and Hamamatsu camera C11440 (Carl Zeiss Microimaging GmbH, Jena, Germany) in a humidified atmosphere containing 5% CO_2_ at 37°C.

For calcium imaging, fibroblasts were stained with 0.1 μ*M* MitoTracker Deep Red FM (Thermo Fisher Scientific) in DMEM+/+ and Fluo4-AM (Thermo Fisher Scientific). During imaging, Fluo4-AM was present in the medium at a 1:1 (v/v) dilution. During imaging, cells were treated with 1 μ*M* thapsigargin (Sigma–Aldrich). To test for the involvement of mitochondria in cytosolic calcium regulation, we incubated the cells with 10 μ*M* Ru360 (Sigma–Aldrich). After treatment, time-laps imaging was continued for 10 min and images were acquired every 2 s.

For analysis of colocalization of mitochondria and LC3 puncta, immortalized fibroblasts were transfected with mito-DsRed (5) and eGFP-LC3 (22) using TransIT-2020 transfection reagent (Mirus Bio, LLC). Cells were then treated with 25 μ*M* CCCP or 10 n*M* bafilomycinA_1_ for 2 or 6 h, respectively.

For analysis of autophagosome formation, cells were stained with 0.2 m*M* 18:1 NBD-PS (810198C; Sigma–Aldrich) for 30 min at 37°C. Cells were subsequently starved in medium without FBS for 2 h.

For colocalization analysis of mitochondria and ER, native fibroblasts were stained with 0.1 μ*M* MitoTracker Deep Red FM and 1 μ*M* ER-Tracker Green (Thermo Fisher Scientific) and imaged using the 368 or 488 nm laser.

For analysis of calcium-induced mitochondrial fragmentation, immortalized fibroblasts were stained with 0.1 μ*M* MitoTracker Green FM (Thermo Fisher Scientific) for 45 min at 37°C. After 1 min of imaging, cells were treated with 20 μ*M* ionomycin (Sigma–Aldrich) and imaging was continued for 20 min.

All image analysis was performed with MATLAB or ImageJ.

### Immunofluorescence stainings

For colocalization analysis of mitochondria and ER, native fibroblasts were fixed with 4% paraformaldehyde for 15 min and then labeled with antibodies against Tom20 (sc-17764, dilution 1:500, secondary antibody:goat anti-mouse Alexa Fluor 647; Santa Cruz Biotechnologies; A-21235, dilution 1:1000; Life Technologies) and PDI (2446S, dilution 1:1000, secondary antibody:goat anti-rabbit Alexa Fluor 488; Cell Signaling Technology; A-1000, dilution 1:1000; Life Technologies).

### Measurement of ATP level

Steady-state ATP levels were measured in immortalized fibroblasts (*n* = 500,000 cells) grown under standard conditions. The quantification of total ATP levels was performed with the ATP Bioluminescence Assay Kit CLS II (Roche) according to the manufacturer's protocol in 96-well plates (Greiner Bio-One). The luminescence signal was detected with the Microplate Reader infinite M200Pro (TECAN).

### Statistics

Statistical significance was determined using the GraphPad Prism 6.0 software. Statistical tests and *p*-values are indicated in the figure legends. To account for the small sample size used in this study, we employed nonparametric tests throughout. All experiments were independently repeated at least three times (*n* represents the number of independent biological replicates).

## Supplementary Material

Supplemental data

Supplemental data

## Abbreviations Used

CCCP: carbonyl cyanide 3-chlorophenylhydrazone
DMEM: Dulbecco's modified Eagle's medium
ER: endoplasmic reticulum
EU: European Union
FBS: fetal bovine serum
LoF: loss-of-function
MCU: mitochondrial calcium uniporter
miRNA: microRNA
MiST: mitochondrial shape transition
o/e: observed/expected
PD: Parkinson's disease
PE: phosphatidylethanolamine
pLI: probability of being LoF intolerant
PS: phosphatidylserine
