# Supplementary material for: Mutations in RHOT1 Disrupt Endoplasmic Reticulum–Mitochondria Contact Sites Interfering with Calcium Homeostasis and Mitochondrial Dynamics in Parkinson's Disease
Source: Antioxid Redox Signal. 2019 Oct 17;31(16):1213–34. doi: 10.1089/ars.2018.7718 (PMC6798875; doi:10.1089/ars.2018.7718)
Supplement: Supplemental data [file Supp_Fig1.pdf]

Supplementary Data

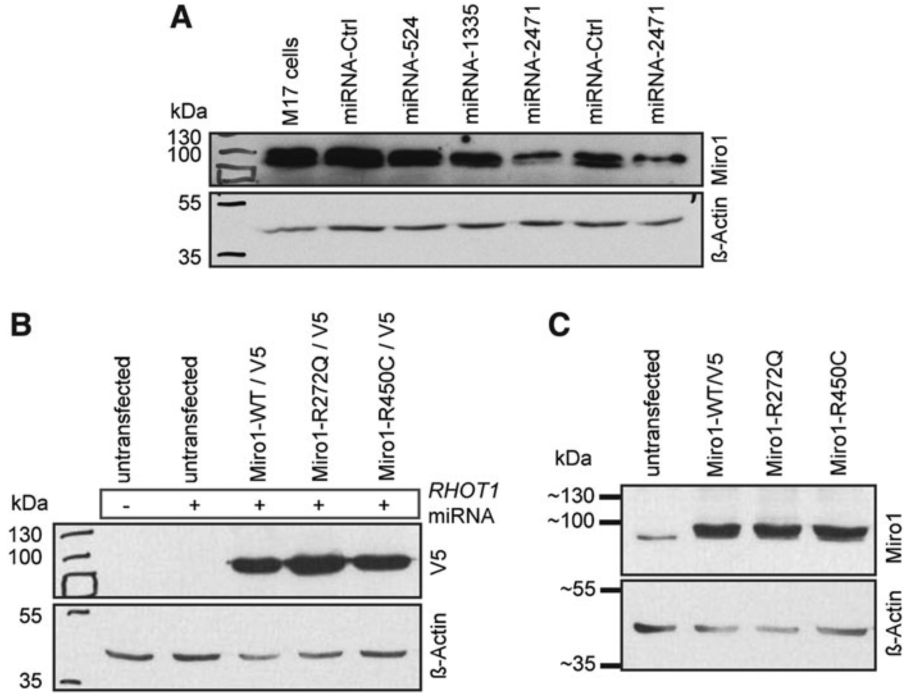

SUPPLEMENTARY FIG. S1. Characterization of knockdown of endogenous *RHOT1* and overexpression of *RHOT1* in M17 cells. (A) A stable knockdown of endogenous *RHOT1* was established in M17 cells by transfection with miRNAs targeting different regions of *RHOT1* and subsequent selection of positively transfected cells with 6 μg/mL blasticidin. Knockdown of *RHOT1* was most efficient by using the miRNA-2471, which is targeting the 5′UTR of *RHOT1*. Therefore, we chose the miRNA-2471 for all further experiments. **(B)** Representative Western blot image of untransfected M17 cells or M17 expressing different variants of Miro1 in the pcDNA3.1/V5-HisA vector: WT Miro1 (Miro1-WT/V5), Miro1-R272Q, and Miro1-R450C. Western blot was probed using an antibody against the V5 tag (R960-25; Sigma–Aldrich). Untransfected cells show no signal for V5. **(C)** Representative Western blot image of untransfected M17 cells or M17 expressing different variants of Miro1. Western blot was probed with an antibody against Miro1 (HPA010687; Sigma–Aldrich). Untransfected M17 cells show a band for endogenous Miro1, whereas M17 cells transfected with the pcDNA3.1/V5-HisA vector containing *RHOT1* variants show a strong band for overexpressed Miro1 protein. miRNA, microRNA; UTR, untranslated region; WT, wild type.
