# Supplementary material for: Mutations in RHOT1 Disrupt Endoplasmic Reticulum–Mitochondria Contact Sites Interfering with Calcium Homeostasis and Mitochondrial Dynamics in Parkinson's Disease
Source: Antioxid Redox Signal. 2019 Oct 17;31(16):1213–34. doi: 10.1089/ars.2018.7718 (PMC6798875; doi:10.1089/ars.2018.7718)
Supplement: Supplemental data [file Supp_Table1.pdf]

Supplementary Table S1. List of Primers Used for the RHOT1 Mutation Screen in Parkinson's Disease Patients

Exon	Exon length (bp)	PCR product (bp)	primer forward (5' > 3')	primer reverse (5' > 3')	Tm
2	59	212	TTG CCA AAT TTG AGC TTA TG	ACA CAT GCC AAA AGA GAC AG	56°C
3	82	230	TTG AGT GAT AAT ATT CAA AGG TGT TT	CCT CTC TGA ACA TTT GTA AAA GC	54°C
4	44	117	CAA TAT GAA ACA TTG ACT TCC TGT C	TTC TAC ACA AAA CCC CAC ACC	61°C
5	54	163	TTG CTA ATT AAG TGC TGT CTA TG	CCA CTG AAA CCA ACT ATA GGG	59°C
6	53	187	GGC TAA GCG CTT GTT AAT TTT C	AAA TTT AAG TGA AGG ATT TAG GGA AG	57°C
7	109	168	CCT TAT TAC AAT GTG CCC TG	AAA TTT TCA AAT CAA ATT ACA AGG	59°C
8	102	378	CAT GTT GTG ATG TCC ACA AAG	ACC AAA ATG TAA CCA TTT AAT AAG C	60°C
9	99	185	CAT TGC TTA TGA AAA TCA CAG	ACG CAA ATG TCT GAA ATA AAG	54°C
10	109	235	GTG GTT TTA ACG CTG GTT TG	CAA TAA AGA CCT TCA CCT TCT GC	57°C
11	121	289	TGC TTT TCT CCT ATG TGA CTC TG	GGT GGG AGG ATC ACT TGA CT	62°C
12	85	209	TGG TGT TAC CTC ACA AAA GAC	TGG AAA ATT ACA GCT AAA GGC	56°C
13	146	2016	TCT AAA CAT TCT GTA TCC TTG TG	TTG TAA CTC TAG AGG AAA GAG GTG	61°C
14	101	198	CCC CAT AAA TAT TCG TTT AGG	AAA TAT CTC TAA TCA TGA GCT GAA C	57°C
15	131	280	GGT GGA ATG GAG GCT TAG TG	CAA TGG CTA TGA AAA CAT TAT GTA AAC	61°C
16	84	195	GCA GAT ATT GAG ATT TTC GGT TC	GAA GCC AAT TTG TTT TCA AAG AG	59°C
17	120	253	TTT TAA ACT TAG GAA GTC CAG GG	CAT TCA GTC ATA GTC TGT AGG AGC	60°C
18	203	393	CTG GAG GAA ATG CTT GGT TC	GTT GCT GAA CAA TTC CTA TGT G	59°C
19	96	178	TTT TAC CTT TGC TTT AAA ACT CTC	CAA CAA AAT CCC ACA GAA G	56°C
20	123	184	TCA ACT CCT GTC CTT CTG TG	GCC TGG TAC CCA GTA AAT AAA G	52°C
21	118	257	CTG GTA TAG GGT TTT CAT ACT AAT C	ATA ATT CTA GCA GAA CTT AAA GCA TTC	56°C

Exon lengths were determined using the Genome browser webpage (https://genome.ucsc.edu/cgi-bin/hgTracks?db=hg19&lastVirtModeType=default&lastVirtModeExtraState=&virtModeType=default&virtMode=0&nonVirtPosition=&position=chr17%3A30469473%2D30552746&hgsid=725100075_KGJ3cA6ASUEwBxgtEWZOdDHUrx2K).

Tm, annealing temperature of the primers.
